# Plasticity of fibroblasts demonstrated by tissue-specific and function-related proteome profiling

**DOI:** 10.1186/1559-0275-11-41

**Published:** 2014-11-21

**Authors:** Astrid Slany, Anastasia Meshcheryakova, Agnes Beer, Hendrik Jan Ankersmit, Verena Paulitschke, Christopher Gerner

**Affiliations:** Faculty of Chemistry, Department of Analytical Chemistry, University of Vienna, Vienna, Austria; Department of Medicine I, Institute of Cancer Research, Medical University of Vienna, Vienna, Austria; Christian Doppler Laboratory for the Diagnosis and Regeneration of Cardiac and Thoracic Diseases, Medical University Vienna, Vienna, Austria; Department of Dermatology, Medical University of Vienna, Vienna, Austria; Department of Thoracic Surgery, Division of Surgery, Medical University Vienna, Vienna, Austria; Christian Doppler Laboratory for the Diagnosis and Regeneration of Cardiac and Thoracic Diseases, Medical University Vienna, Vienna, Austria

**Keywords:** Fibroblasts, Inflammatory activation, Tumor-stroma interactions, Primary human cells, Proteome profiling

## Abstract

**Background:**

Fibroblasts are mesenchymal stromal cells which occur in all tissue types. While their main function is related to ECM production and physical support, they are also important players in wound healing, and have further been recognized to be able to modulate inflammatory processes and support tumor growth. Fibroblasts can display distinct phenotypes, depending on their tissue origin, as well as on their functional state.

**Results:**

In order to contribute to the proteomic characterization of fibroblasts, we have isolated primary human fibroblasts from human skin, lung and bone marrow and generated proteome profiles of these cells by LC-MS/MS. Comparative proteome profiling revealed characteristic differences therein, which seemed to be related to the cell’s tissue origin. Furthermore, the cells were treated *in vitro* with the pro-inflammatory cytokine IL-1beta. While all fibroblasts induced the secretion of Interleukins IL-6 and IL-8 and the chemokine GRO-alpha, other inflammation-related proteins were up-regulated in an apparently tissue-dependent manner. Investigating fibroblasts from tumorous tissues of skin, lung and bone marrow with respect to such inflammation-related proteins revealed hardly any conformity but rather individual and tumor type-related variations. However, apparent up-regulation of IGF-II, PAI-1 and PLOD2 was observed in melanoma-, lung adenocarcinoma- and multiple myeloma-associated fibroblasts, as well as in hepatocellular carcinoma-associated fibroblasts.

**Conclusions:**

Inflammation-related proteome alterations of primary human fibroblasts were determined by the analysis of IL-1beta treated cells. Tumor-associated fibroblasts from different tissue types hardly showed signs of acute inflammation but displayed characteristic functional aberrations potentially related to chronic inflammation. The present data suggest that the state of the tumor microenvironment is relevant for tumor progression and targeted treatment of tumor-associated fibroblasts may support anti-cancer strategies.

**Electronic supplementary material:**

The online version of this article (doi:10.1186/1559-0275-11-41) contains supplementary material, which is available to authorized users.

## Background

Fibroblasts have long been considered as rather simple structural cells contributing to the extracellular matrix (ECM) in connective tissues and responsible for tissue repair during wound healing. In the last years, however, these cells have been recognized to play an important role in other processes as well, being able to exert a significant influence on all kind of cells in their environment. So, it appears now evident that fibroblasts are decisive players in inflammatory processes, modulating the activities of leukocytes by secreting specific signaling molecules at sites of inflammation [1]. Importantly, fibroblasts seem to be responsible for the termination of immune responses, or alternatively, for the switch from acute to chronic inflammation [2, 3]. During acute inflammation, immune cells are recruited and expanded in the damaged tissue. Under normal physiological conditions, control mechanisms prevent from over-stimulating these cells. Hereby fibroblasts play an important role by releasing immune-regulatory signals which promote the removal of dead or redundant immune cells. Chronic inflammation may occur when these clearance mechanisms fail. This may happen when fibroblasts either inhibit apoptotic processes by producing survival cytokines, or retain the immune cells by the release of specific chemokines [2]. Chronic inflammation processes are also involved in diseases such as fibrosis and cancer. Regulatory effects of fibroblasts on the development and progression of fibrosis and cancer have been demonstrated [4–8]. Reorganization of the ECM by fibroblasts plays thereby an important role. In tumors, additionally, interactions between fibroblasts and tumor cells seem to be essential for tumor growth and progression. These interactions are mediated through soluble signaling factors such as growth factors, cytokines, chemokines and lipid products, or by direct communication of the cells through integrins [9] ECM-degrading enzymes such as matrix metalloproteinases (MMPs), produced by fibroblasts, contribute to the degradation and remodeling of the tumor environment, thereby supporting tumor progression, including angiogenesis, invasiveness and metastasis [10].

It appears nowadays clear that fibroblasts are not only involved in structural concerns, but are also important players in patho-physiological processes. However, a lot of questions remain unanswered. So, the characterization of different fibroblast sub-types, including their expression profiles in inactivated as well as in activated and disease-related cell states, is still not clearly assessed. Up until now, no selective markers have been established, and cells with spindle-like morphology, which *in*-*vitro* adhere on plastic ware, and furthermore have non-lymphoid, non-endothelial and non-epithelial phenotypes, are generally considered as fibroblasts [1]. Some attempts have therefore been made to better characterize these cells [4, 11, 12]. However, the results of these investigations are raising new questions. In effect, it seems that more different sub-types of fibroblasts exist than expected [13]. Some researchers even claim to define new cell types and point out the fact that fibroblasts from different anatomic sites display differences which are comparable to those observed among different lineages of leukocytes [14]. The possibility cannot be excluded that multiple sub-types are co-localized at a single location. Furthermore, the origin of fibroblasts at a specific site cannot be accurately determined. They may arise from the primary mesenchyme, or alternatively from BM derived precursor cells, or from local epithelial-mesenchymal transition (EMT) [6, 15]. Knowledge about specific marker molecules, which could distinguish between fibroblasts from different anatomic sites, as well as between fibroblasts at different functional or disease-related states, would massively contribute to the understanding of patho-physiological mechanisms. Furthermore, in cancer, mortality is often related to distant metastases which rather occur as late steps of disease progression. Early cancer markers are therefore urgently needed to allow prompt treatments before aggressive forms of cancer can develop. Such early disease markers could be found in the altered stromal microenvironment of the tumor, whose main contributors are fibroblasts. Characterization of exact phenotypes of normal as well as of activated and disease-related fibroblasts would be of particular importance in this context. As different functionalities are accompanied by the synthesis or up-regulation of specific proteins, they may be revealed by expression or proteome profiling experiments.

The aim of this study was to contribute to the proteomic characterization of fibroblasts from different anatomic sites and at different functional and disease-related cell states. Proteome profiles of normal human lung fibroblasts were generated and compared to those previously obtained from primary skin and BM fibroblasts. The aim was to work out characteristic proteome signatures for these cells. Furthermore, the same cells were treated in an inflammatory way using IL-1β, in order to find out function-specific proteome alterations. Finally, in a last step we aimed to determine the functional states of tumor-associated fibroblasts from analogous tissues. On the one hand the proteome profile of lung cancer-associated fibroblasts was analyzed. On the other hand, previously published proteomic data obtained from the analysis of fibroblasts related to melanoma and thus representing cancer-associated skin fibroblasts were used, as well as fibroblasts obtained from patients with multiple myeloma, a plasma cell tumor of the BM [16, 17]. Furthermore, results obtained from our previous investigations concerning HCC-associated fibroblasts [18] were included in the present study for comparative analyses. The aim was to answer the questions if tumor-associated fibroblasts manifest an inflammatory activated cell state, and if fibroblasts related to different cancers may reveal similarities regarding their proteome profiles.

## Results and discussion

### Experimental strategy

An important aim of this study was to determine marker proteins which would allow distinguishing different phenotypes of fibroblasts. The first objective was to find out if it was possible to discern proteome signatures characteristic for fibroblasts from different tissue origins. Comparative proteome profiling was used to clarify this question. Fibroblasts isolated from different tissues, namely from skin, lung and BM, analyzed by LC-MS/MS based proteome profiling, were compared. Moreover, these cells were analyzed at different functional states, i.e. in non-activated, an inflammatory activated and tumor-associated states. This allowed us to characterize function-related proteome signatures as well. Beside characteristic features of tumor-associated fibroblasts, the inflammatory status of these cells was of special interest.

### Cell culture and characterization of primary cells, as well as treatment of cells with IL-1β

Primary human lung fibroblasts isolated both, from tumor, as well as from non-tumor tissue areas were cultivated for three passages in order to get enough cells for further analyses. Compatible with our observations, Chang *et al*. have demonstrated that fibroblasts maintain a stable phenotype for several passages during cell culture conditions [14]. The cells were then characterized by FACS analysis (Additional file 1: Figure S1), which revealed that they were highly positive for CD90, but negative for leukocyte, endothelial cell and hematopoietic stem cell markers CD45, CD31 and CD34, proving the purity of the cell preparations. Furthermore, the cells showed typical fibroblast’s characteristics, such as adherent growth on culture flasks, as well as a spindle-like morphology. 41.3% and 62.1% of the cells from non-cancerous and cancer-associated areas, respectively, were positive for CD54 (ICAM-1), which indicated inflammatory activation [19] in both kinds of cells, even though more cancer-associated fibroblasts were activated than non-cancerous ones. Furthermore, 34% of cancer-associated fibroblasts were positive for alpha-smooth muscle actin (α-SMA), corresponding to a myofibroblast phenotype characteristic for cancer-associated fibroblasts [20]. However, 21.5% of the fibroblasts from non-cancerous areas showed α-SMA-positive staining as well, indicating a slight amount of α-SMA-positive cells in this cell population. For comparison, we also included cells acquired by purchase, namely NHLF, in our investigations as reference cell system for normal lung fibroblasts. NHLF were used in their non-activated cell state, or alternatively treated for 24 hours with IL-1β in order to generate fibroblasts in an inflammatory activated cell state. Skin- and BM-derived fibroblasts, raised as previously described [17, 21], were treated with IL-1β in the same way.

### Generation of proteome profiles of primary human fibroblasts at different functional cell states

In a first step, proteome profiles of sub-cellular fractions (cytoplasm, secretome and nuclear extract) of NHLF and primary lung fibroblasts were generated. Data of the sub-cellular fractions were afterwards combined in order to obtain comprehensive proteome profiles for each cell type, which resulted in a total of 1577 and 1545 proteins for NHLF and primary lung fibroblasts, respectively (Additional file 2: Table S1 and Additional file 3: Table S2). For comparative analyses, which was supported by our specially designed database, the GPDE (see Methods), the proteome profile of skin fibroblasts, comprising 1496 proteins (Additional file 4: Table S3), as well as that of BM-derived fibroblasts, including 1569 proteins (Additional file 5: Table S4) were further used. Parts of these proteome profiles have been published previously [17, 21]. However, the present protein lists may differ slightly as for comparative analysis we now also included one peptide-identifications when the corresponding protein was identified in any other of the here investigated samples with two or more peptides.

Proteome profiles of the inflammatory activated cells were generated similarly, identifying 1608 proteins in activated NHLF (Additional file 6: Table S5), 1428 proteins in activated skin fibroblasts (Additional file 7: Table S6), and 1347 proteins in activated BM fibroblasts (Additional file 8: Table S7). Finally, for the consideration of tumor-associated fibroblasts we again made use of previously published proteome profiles obtained from the analysis of melanoma-associated skin fibroblasts [16], multiple myeloma-associated BM fibroblasts [17] and HCC-associated fibroblasts [18]. The respective proteome profiles are summarized in Additional file 9: Table S9, Additional file 10: Table S10, Additional file 11: Table S11. In addition, proteome profiles of lung cancer-associated fibroblast were analyzed, which resulted in a total of 1454 identified proteins (Additional file 12: Table S8).

### Primary human fibroblasts from different tissue types display differences in their proteome profiles

**Figure 1:**
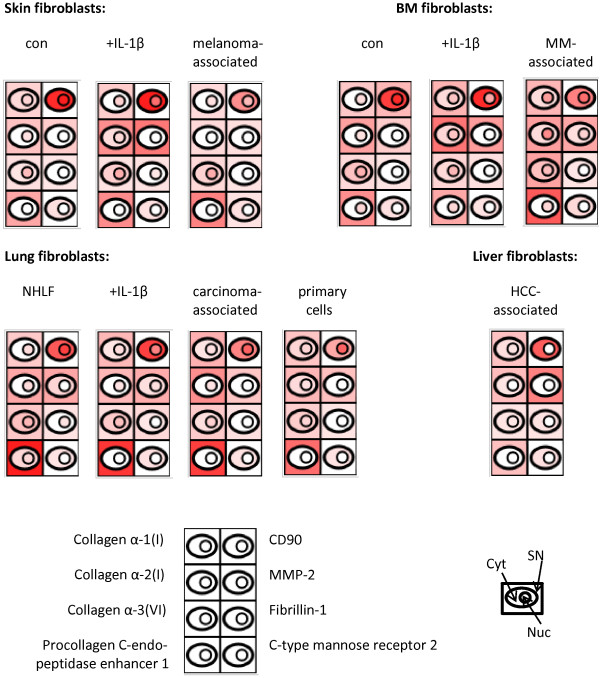
**Proteins which are found in all kinds of fibroblasts.** Based on the proteome profiles of skin, bone marrow (BM), lung and liver fibroblasts in different functional states, a semi-quantitative assessment of selected proteins in these cells was performed using the corresponding emPAI values (see Methods). The emPAI values for all sub-cellular fractions are visualized by colored cell symbols, increased color intensities corresponding to increased protein abundances. Con, non-activated control cells; +IL-1β, IL-1β treated cells; Cyt, cytoplasmic fraction; SN, cell supernatant; Nuc, nuclear fraction; NHLF, normal human lung fibroblasts; MM, derived from multiple myeloma patients; HCC, derived from hepatocellular carcinoma patients.

Stromal fibroblasts have several common biological tasks irrespective of the tissue origin or functional state of the cells. Such tasks include generation and maintenance of the ECM and provision of physical support and elasticity, and may be related to specific protein synthesis. A similarity in the proteome profiles regarding proteins related to such functions should therefore be observed in fibroblasts, irrespective of their tissue origin. Indeed, several ECM-related proteins such as fibrillin-1 and different subtypes of collagens were identified by us in all fibroblasts isolated from skin, lung and BM, irrespective of the functional states of the cells (Figure 1). Furthermore, in all kinds of fibroblasts we further detected ECM modulating enzymes such as procollagen C-endopeptidase enhancer 1, which contributes to the cross-linking of ECM proteins [22], as well as MMP-2 and c-type mannose receptor 2, which are both involved in collagen degradation, as described previously [17]. CD90, procollagen C-endopeptidase enhancer 1, and c-type mannose receptor 2 were listed in the proteome signature specific for fibroblasts, published recently [21].

**Figure 2:**
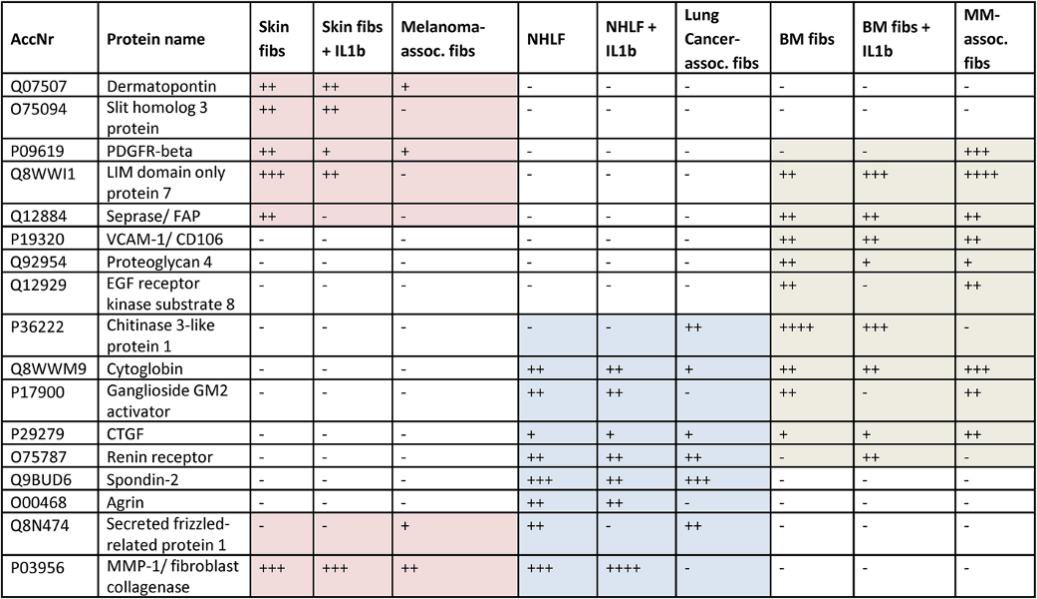
**Protein signatures characteristic for fibroblasts of different tissue origins.** The total number of distinct peptides identified per protein when considering all three sub-cellular fractions was used as a semi-quantitative measure for protein abundances in the different fibroblasts. AccNr, Swiss-Prot accession number; -, protein was not detected; +, protein was detected with one peptide; ++, protein was detected with 2 to 5 peptides; +++, protein was detected with ≥6 peptides; ++++, protein was detected with ≥15 peptides.

Beside such common features characteristic for all types of fibroblasts, we expected some functional specialization of the cells, related to the tissue origin. Therefore, the data obtained from skin, lung and BM fibroblasts, comprising all data from control, inflammatory activated and tumor-associated fibroblasts were searched for proteome signatures potentially characteristic for the fibroblast’s tissue origin. Comparative proteome profiling searching for proteins detected exclusively in fibroblasts of one tissue origin suggested the existence of tissue-specific signatures. Taking the close relation of cell functions into account, proteins detected in fibroblasts from two kinds of tissue origins but absent from the third tissue were considered as well. All selected proteins were verified regarding their biological functionalities and regarding known literature about tissue specificity. The finally compiled signatures, each potentially characteristic for fibroblasts of one tissue type are listed in Figure 2, with corresponding emPAI values listed in Additional file 13: Table S12. We thus suggest that the tissue of origin of fibroblasts may be determined making use of such signatures; the practical applicability thereof needs to be established in the future.

The presently suggested signature characteristic for skin fibroblasts contained dermatopontin, slit homolog 3 protein, PDGFR-beta, LIM domain only protein 7, seprase, secreted frizzled-related protein 1, and MMP-1 (Figure 2, rose colored). Dermatopontin is an abundant dermal extracellular matrix protein, contributing to the communication of dermal fibroblasts with their environment [23]. Slit homolog 3 protein plays an important role as general cell migration guidance molecule, supporting epidermal cell migration [14]. Remarkably, Denk *et al*. have observed that this protein may be hindering the migration of malignant melanoma cells [24]. Compatible with this notion, an apparent down-regulation of this protein in melanoma-associated skin fibroblasts was observed. An intriguing idea might be that melanoma cells may influence neighboring fibroblasts in terms of down-regulation of this protein which would otherwise restrict their motility. PDGFR-beta was detected by us in all kinds of skin fibroblasts, as well as in in multiple myeloma-associated BM fibroblasts, which we have discussed previously [17]. The observation that BM fibroblasts up-regulate this protein only when they are in a proliferation state was further supported by others [25]. PDGFR-beta has also been described to occur in lung fibroblasts [26], however, the protein was not detected in such cells analyzed by us. MMP-1 was detected by us in all kinds of skin fibroblasts and in control as well as in inflammatory activated lung fibroblasts, but not in BM-derived cells. Halfon *et al*. have described MMP-1 as one of the proteins which may be a suitable marker for skin and lung fibroblasts allowing distinguishing them from BM-derived mesenchymal stromal cells [27], which is compatible with the present interpretation. Adenocarcinoma-related cells apparently express lower amounts of MMP-1 than other lung cancers [28], which may explain why MMP-1 was not detected by us in cancer-associated lung fibroblasts.

The presently suggested signature characteristic for BM-derived fibroblasts included PDGFR-beta, LIM domain only protein 7, seprase, V-CAM-1, proteoglycan 4, EGF receptor kinase substrate 8, chitinase 3-like protein 1, cytoglobin, ganglioside GM2 activator, as well as CTGF and renin receptor (Figure 2, green colored). VCAM-1 is known since long to be produced by BM fibroblasts [29]. Proteoglycan 4 has been described to be predominantly synthesized by BM stromal cells, regulating immune cells and parathyroid hormone actions on BM hematopoietic progenitor cells [30, 31]. Seprase (also called fibroblast activation protein alpha) has been proposed as candidate marker protein for BM mesenchymal stromal cells by Bae *et al*. [32]. This protein was detected by us in all kinds of BM fibroblasts, but also in control skin fibroblasts. LIM domain only protein 7 was similarly detected in all kinds of BM fibroblasts, the up-regulation observed in inflammatory activated and tumor-associated BM fibroblasts correlating with that observed in breast cancer-associated stromal cells; the protein seems to be important for cancer cell migration [33]. One of the proteins detected in BM-fibroblasts and NHLF, but not in skin fibroblasts, cytoglobin, has similarly been described by Nakatani *et al*. to be present in several fibroblasts, but not in dermal ones [34]. Chitinase 3-like protein 1, previously described by us [17], was found to be down-regulated in tumor-associated BM fibroblasts. In lung cancer-associated fibroblasts, on the other hand, it was found by us to be up-regulated relative to control cells, which was supported by observations of others [35].

Finally, the presently suggested proteome signature characteristic for lung-derived fibroblasts comprised chitinase 3-like protein 1, cytoglobin, ganglioside GM2 activator, CTGF, renin receptor, spondin-2, agrin, secreted frizzled-related protein 1, and MMP-1 (Figure 2, blue colored). Actually, spondin-2, together with secreted frizzled-related protein 1, is required for lung morphogenesis. These two proteins have antagonistic effects on the Wnt signaling pathway which is critical in the induction of cell differentiation necessary for the formation of organs such as the lung [36, 37]. Agrin has also been described in the context of lung, apparently being up-regulated in the basement membranes of adult lungs, probably playing a role as mediator in pulmonary function [38]. Concerning renin receptor, this protein may be involved in the renin-angiotensin system, which is regulating the blood pressure, but seems also to play a role in lung cell inflammation and proliferation [39, 40].

### Primary human fibroblasts from different tissue origins display differences in their inflammatory signature

**Figure 3:**
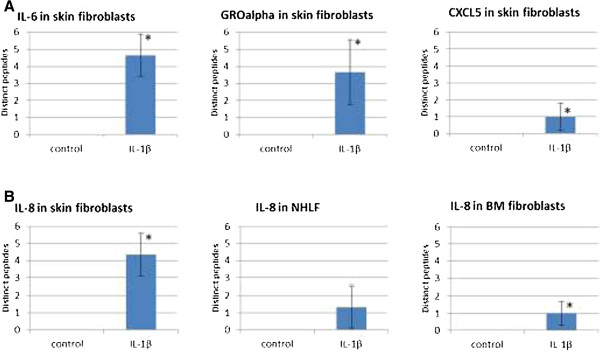
**Up**-**regulation of selected proteins in the cell supernatant of fibroblasts upon IL-1β treatment.** Successful inflammatory activation in fibroblasts upon IL-1β treatment was demonstrated by up-regulation of known inflammation-induced proteins. The mean number of distinct peptides identified per protein in the respective cell supernatants, determined from at least three independent experiments, was used. Significant up-regulation was determined using the Chi-squared test (*, p <0.05). **(A)** Up-regulation of IL-6, GRO-alpha and CXCL5 in IL-1β treated skin fibroblasts. **(B)** Up-regulation of IL-8 in IL-1β treated skin, lung (NHLF) and BM fibroblasts.

In order to find out characteristics related to an inflammatory activated state of fibroblasts, proteome profiles of lung, skin and BM fibroblasts treated with IL-1β were generated (Additional file 6: Table S5, Additional file 7: Table S6 and Additional file 8: Table S7) and compared with those of the corresponding control cells (Additional file 2: Table S1, Additional file 4: Table S3 and Additional file 5: Table S4). The efficiency of inflammatory activation in response to stimulation with IL-1β was confirmed by the detection of induced proteins known to be up-regulated during an inflammatory response. So, IL-6 and IL-8, classic inflammatory cytokines, were readily identified in all fibroblasts upon inflammatory activation, just as GRO-alpha (CXCL1) and CXCL6, both chemotactic for neutrophils (Table 1, and corresponding emPAI values in Additional file 14: Table S13; Figure 3). Thus, all these fibroblasts proved to be prepared to respond to an inflammatory stimulus and reacted promptly to the IL-1β treatment. Two other proteins, PLOD-2 and palladin were likewise found to be up-regulated in all three kinds of inflammatory activated cells. PLOD-2 is necessary for cross-linking collagens. Up-regulation of the protein was described to occur during inflammatory activation of skin and lung fibroblasts [41, 42]. Furthermore, in several fibrotic diseases, which are characterized by chronic inflammation and by the deposition of ECM components such as collagenous fibers, PLOD-2 seems to be closely involved in disease progression. Wu *et al*. have demonstrated that in dermal fibrosis, PLOD-2 up-regulation also leads to an overexpression of collagen alpha-1(I) which contributes to the development of this skin disease [43]. In the BM, PLOD-2 overexpression has been observed to account for the progression of primary myelofibrosis [44]. Concerning palladin, this protein has been described to be up-regulated in the leading edge of wounds [45]. Apparently, it seems to be involved in wound healing processes, accompanying TGF-beta-1 induced myofibroblast differentiation, and is possibly necessary for the generation of contractile forces in these cells and the transmission of these forces to the ECM [46].

**Table 1:** **Proteins differentially regulated in different fibroblasts upon inflammatory activation**

AccNr	Protein name	Skin fibroblasts		NHLF		BM fibroblasts	
Supernatant:		Control	IL-1β-treated	Control	IL-1β-treated	Control	IL-1β-treated
P05231	IL-6	_	+++	_	++	_	+++
P10145	Il-8	_	+++	_	++	_	++
P09341	GRO-alpha/CXCl1	_	++	_	+++	_	++
P80162	CXCL6	_	++	_	++	_	++
P42830	CXCL5	_	++	_	++	_	_
P08254	MMP-3/Stromelysin-1	_	+++	_	+++	_	_
P07585	Decorin	++	++++	+++	++++	++	++
P08253	MMP-2	++	++++	+++	+++	++	+++
P09603	CSF-1	_	++	++	++	_	++
P98066	TSG-6	_	++	_	_	_	++
P26022	PTX3/ TSG-14	+++	+++	_	++	+++	++++
P19875	GRO-beta /CXCL2	_	++	_	_	_	_
P05121	PAI-1	+++	+++	++	+++	+++	+++
O00391	Sulfhydryl oxidase 1	(+)	++	++++	+++	_	++
Q15063	Periostin	(+)	_	++	+++	(+)	++
Q9Y240	SCGF	++	_	(+)	++	_	_
**Cytoplasm**:							
O00469	PLOD-2	(+)	++	_	+++	(+)	+++
P05362	ICAM-1 (CD54)	_	_	(+)	+	_	++
P05120	PAI-2	++	+++	_	+++	++	_
**Nuclear extract**:							
Q8WX93	Palladin	++	+++	++	+++	++	+++
P51911	Calponin-1	+++	+++	++	+++	++	+++
Q16666	γ-IFN-inducible protein 16	++	++	++	++	++	+++

The total number of distinct peptides identified per protein when considering all three sub-cellular fractions was used as a semi-quantitative measure for protein abundances in the different fibroblasts, which allowed determining up- or down-regulation of proteins upon inflammatory activation. Most of the regulated proteins were found in the secretomes, listed under “Supernatant”.

AccNr, Swiss-Prot accession number; -, protein was not detected; +, protein was detected with one peptide; ++, protein was detected with 2 to 5 peptides; +++, protein was detected with ≥6 peptides; ++++, protein was detected with ≥15 peptides; (+), protein identification was ambiguous, i.e. protein was not found robustly in all corresponding samples.

In addition, apparent tissue-related differences in the responses to the inflammatory stimulus (Table 1) were also observed. Actually, some of the proteins were found to be up-regulated only in one type of fibroblasts. So, GRO-beta (CXCL2) was only detected in activated skin fibroblasts, PAI-1 in activated lung fibroblasts, and γ-IFN-inducible protein 16 in activated BM fibroblasts. This latter protein, even though also described to be involved in senescence of fibroblasts [47], is considered primarily to play an important role in inflammatory processes. Indeed, it seems to be a critical, predominantly nuclear sensor for detecting pathogenic DNA and inducing pro-inflammatory responses [48]. In endothelial cells, the protein has been described to be necessary for IFN-γ triggered stimulation of pro-inflammatory genes through NF-κB, which apparently leads to the up-regulation of adhesion molecules such as ICAM-1, as well as chemokines and cytokines such as IL-8 and CXCL6 [49]. We found up-regulation of this protein in IL-1β treated BM-derived fibroblasts, showing that fibroblast as well may be able to respond to an inflammatory stimulus through γ-IFN-inducible protein 16.

Other proteins were found by us to be up-regulated by IL-1β in two kinds of fibroblast, such as MMP-3 in activated skin and lung fibroblasts, MMP-2 in activated skin and BM fibroblasts and ICAM-1 in activated lung and BM fibroblasts. Furthermore, three proteins displayed oppositional modes of regulation. So, PAI-2 was found to be up-regulated in activated skin and lung fibroblasts, but down-regulated in activated BM fibroblasts; sulfhydryl oxidase 1 was apparently up-regulated in activated skin and BM fibroblasts, but down-regulated in activated lung fibroblasts; and SCGF was up-regulated in activated lung fibroblasts, down-regulated in activated skin fibroblasts, and not detected by us in BM fibroblasts. The majority of these proteins can immediately be related to an inflammatory related mechanism, most of them being induced through IFN-γ and NFκB [50–55]. Therefore, our observations may suggest tissue-type specific modulation of inflammation-related processes.

### Characterization of tumor-associated fibroblasts _ PLOD-2 and PAI-1 as possible markers for chronic inflammation and IGF-II as potential marker for tumor-associated fibroblasts

**Figure 4:**
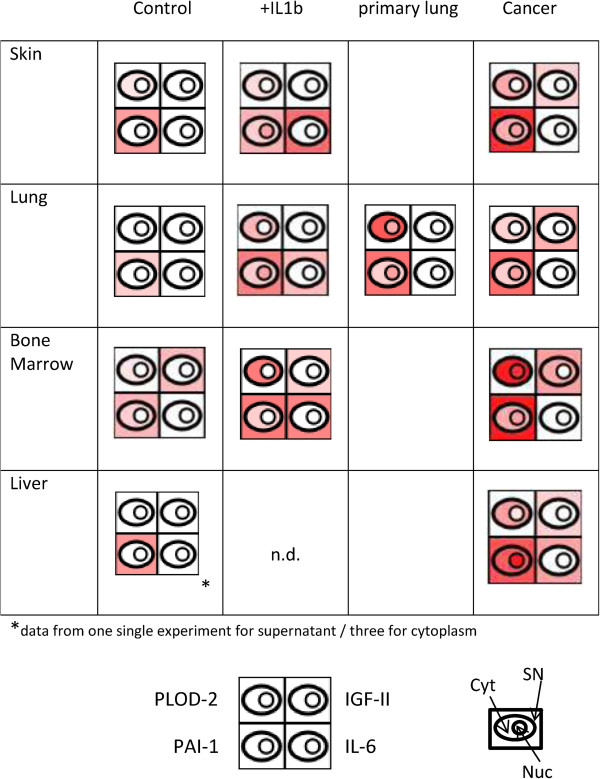
**Proteins characteristic for inflammatory activated and/or tumor-associated fibroblasts.** Based on the proteome profiles of skin, bone marrow (BM), lung and liver fibroblasts in different functional states, a semi-quantitative assessment of selected proteins in these cells was performed as described for Figure 1. Primary lung, primary lung fibroblasts isolated from non-cancerous tissue areas; tumor, fibroblasts isolated from tumor tissue areas.

As fibroblasts seem to be highly involved in chronic inflammatory processes, and as further chronic inflammation seems to play an important role for tumor development, we wanted to assess the inflammation status of tumor-associated fibroblasts. Tumor-associated fibroblasts related to skin, lung, BM, as well as liver, namely from melanoma, lung adenocarcinoma, multiple myeloma and HCC were analyzed. For HCC it is well-known that acute as well as chronic inflammatory processes play an important role in disease progression [56]. Actually, in HCC-associated fibroblasts we found up-regulation of the classical inflammation mediators, IL-6, IL-8 and GRO-alpha, which are proteins up-regulated in the early phase of inflammation. Those were not detected in any other tumor-associated fibroblasts, as demonstrated for IL-6 in Figure 4. However, in all kinds of tumor-associated fibroblasts we observed up-regulation of PAI-1 and PLOD-2 (Figure 4). PLOD-2 has been described to be up-regulated in several fibrotic diseases characterized by chronic inflammation, as mentioned above. Interestingly, PLOD-2 has recently been proposed as a marker protein for different cancer-associated fibroblasts by a method based on RNA profiling data [57]. Moreover, PLOD-2 has also been described as negative prognostic factor for HCC progression [58]. PAI-1 has also been demonstrated to play an important role in fibrotic processes. It was suggested that the pro-fibrotic effect of PAI-1 results from the ability of this protein to recruit increasing numbers of macrophages and myofibroblasts to fibrotic tissues [59]. Therefore, these two proteins may be indicators for a chronic inflammatory status in tumor-associated fibroblasts.

Apart from proteins indicating an activated inflammatory state of the tumor-associated fibroblasts, we also identified IGF-II, a known tumor promoter [60–62], in all kinds of tumor-associated fibroblasts (Figure 4). In case of tumor-associated fibroblasts of skin, lung and liver, identification of IGF-II was patient-dependent. This may reflect the fact that we are working with primary cells, which represent biological material prone to individual variability. However, in case of the liver, up-regulation of IGF-II was already observed by others [63]. In tumor-associated BM fibroblasts, IGF-II was robustly detected by us in all samples, compatible with the notion that IGF-II contributes to multiple myeloma tumor cell growth [64]. IGF-II was apparently constitutively synthesized in the tumor-associated BM fibroblasts, because the protein was detectable not only in the supernatant, but also in the cytoplasm of these cells (Figure 4). That IGF-II was detected in control and IL-1β treated cells as well, although in lower amounts, may reflect the fact that BM fibroblasts are closely related to BM mesenchymal stem cells, which have been described to be characterized precisely by the expression of markers such as IGF-II [27]. A possible explanation for the appearance of IGF-II in the proteome profiles of tumor-associated skin and lung fibroblasts could also be that these fibroblasts may at least partly be derived from freshly recruited BM precursor cells. Actually, it is not yet clear whether tumor-associated fibroblasts arise from local fibroblasts, from the BM or rather result from local EMT [6, 15].

Interestingly, the primary lung fibroblasts, obtained from non-cancerous lung tissue areas well beside tumors, were likewise characterized by high amounts of PLOD-2 and PAI-1 (Figure 4), whereas no IGF-II was detectable in these cells. Our FACS analysis data further have revealed that 21.5% of these cells were positive for α-SMA, which is a marker for cancer-associated fibroblasts. It could therefore be that these fibroblasts were already part of a reactive stromal microenvironment, favorable for the further progression of the cancer [4, 65]. Thus, the data may suggest that up-regulation of PLOD-2 and PAI-1 in fibroblasts is involved in chronic inflammatory processes which are favorable for the early phase of tumor development, whereas induction of IGF-II may play an important role in promoting tumor progression.

## Conclusions

Comparative analysis of primary human cells allowed us to present protein signatures apparently characteristic for fibroblasts originating in skin, lung and BM. Furthermore, we have determined inflammation-related proteome alterations in these cells. When analyzing tumor-associated fibroblasts from clinical tissue samples, substantial individual variations were apparent. However, characteristic functional aberrations were indicated by up-regulation of IGF-II, PAI-1 and PLOD-2. Monitoring such candidate markers could provide important information about the state of the tumor microenvironment of individual samples, independent of the tumor type. Using such information may support the development of improved anti-cancer strategies.

## Methods

### Cell isolation and cell culture conditions

All kinds of fibroblasts were grown in fibroblast basal medium (FBM; Lonza Clonetics, #CC-3131) supplemented with one FGM BulletKit (Lonza Clonetics, #CC-3130) and 10% FCS, in a humidified 37°C-tempered atmosphere containing 5% CO_2_. Normal human lung fibroblasts (NHLF) were purchased from Lonza (Lonza Clonetics, #CC-2512). In parallel, primary human fibroblasts were isolated from lung biopsies of patients suffering from lung adenocarcinoma, either from non-cancerous, or from cancer-associated tissue areas. Each tissue sample was treated for five hours with 200 u/ml of collagenase IV (Eubio, Wothington Biochemicals, #LS004188), and then placed into a culture flask containing FBM (including one FGM BulletKit and 10% FCS) and incubated at 37°C/5% CO_2_ for about five days until fibroblasts grew out of the tissue. Tissue material was then removed and fibroblasts were allowed to grow until reaching 75% of confluence. All samples were obtained with written consent of the respective donor and the approval of the Ethics Committee of the Medical University of Vienna. Human skin fibroblasts were isolated from skin punches and BM fibroblasts from BM aspirates as described previously [17, 21]. For each cell type, at least three biological replicates, corresponding to approximately 10^6^ cells each, were prepared and subjected to the subsequent sample processing steps. Fibroblasts obtained from lung biopsies were characterized by FACS analysis prior to further processing.

### FACS analysis

FACS analysis was performed as previously described [17]. Shortly, primary human lung fibroblasts were collected when they had reached approximately 75% of confluence. 5 × 10^4^-5 × 10^5^ cells were resuspended in 50 μl PBS and unspecific binding was blocked using Beriglobin (CSL Behring; diluted 1:8 in PBS/BSA/Azid). 10 μl directly fluorochrome-conjugated antibody was added and after washing and fixing the cells, they were subjected to flow cytometric analysis using a LSRII instrument (BD Biosciences) and the FlowJo software (TreeStar). Following murine monoclonal antibodies (mABs) were used: FITC-conjugated mAbs specific for alpha-smooth muscle actin (Sigma, #F3777, 1:50), CD45 (Acris, #SM3025F, 1:5), CD31 (Acris, #BM4047F, 1:5) and CD34 (BD Pharmingen, #555821, 1:5); phycoerythrin (PE)-conjugated mAbs specific for CD90 (Eubio, #SM1170R, 1:3) and CD54 (BD Pharmingen, #555511, 1:20).

### Inflammatory activation of fibroblasts

For inflammatory activation, NHLF were treated for 24 hours with IL-1β. Cells were cultivated in FBM (including one FGM BulletKit and 10% FCS), supplemented with 10 ng/ml IL-1β (Sigma-Aldrich, #I9401). After that, cells were further cultivated for 24 hours, now in FCS-free EBM-2 medium, as described in *Cell fractionation*, in order to obtain serum-free cell supernatants. Skin- and BM-derived primary fibroblasts, raised as previously described, [17, 21] were treated with IL-1β in the same way.

### Cell fractionation

Cell fractionation was performed as previously described [17]. In short, cells were washed with EBM-2 (Lonza) without FCS and then cultivated for 24 hours in this serum-free medium to obtain the fraction of secreted proteins without contamination of high-abundant serum proteins coming from FCS. Supernatants were sterile filtered (0.2 μm, FP POINT 2-S, Schleicher & Schuell, Whatman) and ethanol precipitated. For the isolation of cytoplasmic proteins cells were lysed in lysis buffer (10 mM HEPES/NaOH, pH 7.4, 0.25 M sucrose, 10 mM NaCl, 3.5 mM MgCl_2_, 0.5% Triton X-100, 1 mM EGTA; protease inhibitors) and fibroblasts were pressed 12 times through a 23 g syringe to induce cell lysis [66]. The cytoplasmic proteins were separated from nuclei by down-centrifuging the nuclei for 5 minutes at 2300 g and were subsequently ethanol precipitated. To obtain the nuclear extract, the remaining pellet was lysed with 100 mM Tris/HCl pH 7.4, 1 mM EDTA pH 7.5, 500 mM NaCl and afterwards diluted in 10 mM Tris/HCl pH 7.4, 1 mM EDTA pH7.5, 0.5% NP-40 (including protease inhibitors). After centrifugation at 2300 g for 5 minutes, the proteins in the resulting supernatant were ethanol precipitated. Afterwards, proteins of all fractions were pelletized by centrifugation for 20 minutes at 4750 g at 4°C and dissolved in sample buffer (7.5 M urea, 1.5 M thiourea, 4% CHAPS, 0.05% SDS, 100 mM DDT).

### SDS-PAGE for subsequent shotgun analysis

50 μg proteins of each fraction were loaded on a 12% polyacrylamid gel. Electrophoresis was performed until complete separation of a pre-stained molecular marker (Dual Color, Biorad, Hercules, CA). Proteins in the gels were fixed with 50% methanol/10% acetic acid for 30 minutes and silver stained as described [67]. Lanes were then cut into 6 to 8 slices of different molecular weights, and proteins digested with trypsin as described below.

### Digestion with trypsin

The digestion with trypsin was performed as described before [68]. In brief, proteins in gel slices were destained, reduced with DTT and alkylated with iodacetamide before they were digested with trypsin (sequencing grade, Roche) overnight at 37°C. After elution, the peptides were forwarded to LC-MS/MS analysis.

### Mass spectrometry

Mass spectrometry was performed as described previously [66]. In short, peptides were separated by nano-flow LC using the HPLC-Chip technology from Agilent, equipped with a 40 nl Zorbax 300SB-C18 trapping column and a 75 μm x 150 mm Zorbax 300SB-C18 separation column. For peptide elution a gradient from 0.2% formic acid and 2% ACN to 0.2% formic acid and 40% ACN over 60-80 minutes was applied. Peptide identification was accomplished by MS/MS analysis with an iontrap mass spectrometer (XCT-Ultra, Agilent) equipped with an orthogonal nanospray ion source. The MS/MS data were interpreted by the Spectrum Mill MS Proteomics Workbench software (Version A.03.03, Agilent) searching against the SwissProt/UniProtKB protein database for human proteins (Version 12/2010 containing 20328 entries). Peptides were included in the result files when their SpectrumMill score was above 13. Peptides scoring between 9 and 13 were also included if precursor m/z value, retention time and MS2 pattern matched to a reference spectrum scoring above 13. This corresponded to a false discovery rate of less than 1% as described previously [17]. Furthermore, only proteins which were identified in at least one of the cell types with at least two distinct peptides were included in the result files. Concerning protein inference, the smallest number of proteins necessary to explain all observed peptides as described for ProteinProphet [69] was chosen.

### Data interpretation

Data interpretation was supported by the Griss proteomics database engine (GPDE) [70, 71]. The GPDE software can be downloaded freely from http://www.ebi.ac.uk/pride/legacy/. A semi-quantitative assessment of protein abundance was achieved by using the number of distinct peptides identified per protein, as described previously [72]. Alternatively, a semi-quantitative assessment was achieved by determination of the average “emPAI” (exponentially modified protein abundance index) value of an identified protein, according to Ishihama *et al*. [73]. Limits for peptide identification were thereby set from 500 Da to 4500 Da. Furthermore, in order to visualize the abundances of a protein in the different fibroblasts, the emPAI values were transformed into red tones, increased color intensities corresponding to increased emPAI values. We used colored cell symbols for each selected protein; cytoplasm, nucleus and supernatant were colored according to the emPAI values determined for the respective fractions (see Figures 1 and 4). Successful inflammatory activation of fibroblasts was demonstrated by the up-regulation of several proteins known to be induced during an inflammatory response (Table 1). Significance of the up-regulation was determined using chi-squared tests for IL-6, GRO-alpha and CXCL5 in IL-1β treated skin fibroblasts, as well as for one protein (IL-8) in IL-1β treated skin, lung and BM fibroblasts (Figure 3). The significance level therefor was set to 95%.

## Electronic supplementary material

Additional file 1: Figure S1: FACS analysis of primary lung fibroblasts obtained from non-cancerous and cancerous tissue areas. Cells were characterized by FACS analysis, which showed that cells were positive for fibroblast-specific markers CD90, but negative for leukocyte, endothelial cell and hematopoietic stem cell markers CD45, CD31 and CD34 respectively. All samples contained cells which were inflammatory activated, as demonstrated by positive CD54-staining. A certain amount of the cells showed also a positive staining for α-SMA, characterizing a myofibroblast phenotype of cancer-associated fibroblasts. (DOCX 1 MB)

Additional file 2: Table S1: Proteome profile of NHLF. Accession, Swiss-Prot accession numbers; name, protein names; peptides, the number of distinct peptides identified for each protein. (XLSX 102 KB)

Additional file 3: Table S2: Proteome profile of primary lung fibroblasts. Accession, Swiss-Prot accession numbers; name, protein names; peptides, the number of distinct peptides identified for each protein. (XLSX 99 KB)

Additional file 4: Table S3: Proteome profile of skin fibroblasts. Accession, Swiss-Prot accession numbers; name, protein names; peptides, the number of distinct peptides identified for each protein. (XLSX 106 KB)

Additional file 5: Table S4: Proteome profile of BM fibroblasts. Accession, Swiss-Prot accession numbers; name, protein names; peptides, the number of distinct peptides identified for each protein. (XLSX 95 KB)

Additional file 6: Table S5: Proteome profile of NHLF treated with IL-1β. Accession, Swiss-Prot accession numbers; name, protein names; peptides, the number of distinct peptides identified for each protein. (XLSX 90 KB)

Additional file 7: Table S6: Proteome profile of skin fibroblasts treated with IL-1β. Accession, Swiss-Prot accession numbers; name, protein names; peptides, the number of distinct peptides identified for each protein. (XLSX 117 KB)

Additional file 8: Table S7: Proteome profile of BM fibroblasts treated with IL-1β. Accession, Swiss-Prot accession numbers; name, protein names; peptides, the number of distinct peptides identified for each protein. (XLSX 123 KB)

Additional file 9: Table S9: Proteome profile of melanoma-associated fibroblasts. Accession, Swiss-Prot accession numbers; name, protein names; peptides, the number of distinct peptides identified for each protein. (XLSX 104 KB)

Additional file 10: Table S10: Proteome profile of multiple myeloma-associated fibroblasts. Accession, Swiss-Prot accession numbers; name, protein names; peptides, the number of distinct peptides identified for each protein. (XLSX 97 KB)

Additional file 11: Table S11: Proteome profile of HCC-associated fibroblasts. Accession, Swiss-Prot accession numbers; name, protein names; peptides, the number of distinct peptides identified for each protein. (XLSX 104 KB)

Additional file 12: Table S8: Proteome profile of lung carcinoma-associated fibroblasts. Accession, Swiss-Prot accession numbers; name, protein names; peptides, the number of distinct peptides identified for each protein. (XLSX 97 KB)

Additional file 13: Table S12: emPAI values for all proteins listed in Figure 2. For each protein the emPAI values determined by us in the different sub-cellular fractions (sn, cell supernatant; cyt, cytoplasmic fraction; nuc, nuclear fraction) of the respective cell type and cell state are indicated. AccNr, Swiss-Prot accession number. (DOCX 18 KB)

Additional file 14: Table S13: emPAI values for all proteins listed in Table 1. For each protein the emPAI values determined by us in the different sub-cellular fractions (sn, cell supernatant; cyt, cytoplasmic fraction; nuc, nuclear fraction) of the respective cell type and cell state are indicated. AccNr, Swiss-Prot accession number. (DOCX 22 KB)

## Abbreviations

BM: Bone marrow
ECM: Extracellular matrix
EMT: Epithelial-mesenchymal transition
FBM: Fibroblast basal medium
FCS: Fetal calf serum
FITC: Fluorescein isothiocyanate
GPDE: Griss proteomics database engine
HCC: Hepatocellular carcinoma
IGF-II: Insulin-like growth factor II
IL-1β: Interleukin-1beta
LC-MS/MS: Liquid chromatography-tandem mass spectrometry
MMP: Matrix metalloproteinase
NHLF: Normal human lung fibroblasts
PAI-1: Plasminogen activator inhibitor 1
PDGFR-β: beta-type platelet-derived growth factor
PLOD-2: Procollagen-lysine,2-oxoglutarate 5-dioxygenase 2
PRIDE: Proteomics identification database (http://www.ebi.ac.uk/pride).
