# Supplementary material for: Plasticity of fibroblasts demonstrated by tissue-specific and function-related proteome profiling
Source: Clin Proteomics. 2014 Nov 21;11(1):41. doi: 10.1186/1559-0275-11-41 (PMC4448269; doi:10.1186/1559-0275-11-41)
Supplement: Supplementary file 1 — Additional file 1: Figure S1: FACS analysis of primary lung fibroblasts obtained from non-cancerous and cancerous tissue areas. Cells were characterized by FACS analysis, which showed that cells were positive for fibroblast-specific markers CD90, but negative for leukocyte, endothelial cell and hematopoietic stem cell markers CD45, CD31 and CD34 respectively. All samples contained cells which were inflammatory activated, as demonstrated by positive CD54-staining. A certain amount of the cells showed also a positive staining for α-SMA, characterizing a myofibroblast phenotype of cancer-associated fibroblasts. (DOCX 1 MB) [file 12014_2014_89_MOESM1_ESM.docx]

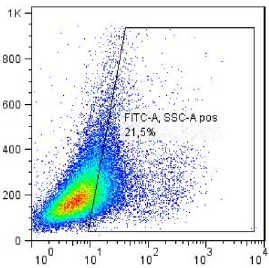
**Control lung fibroblasts:**


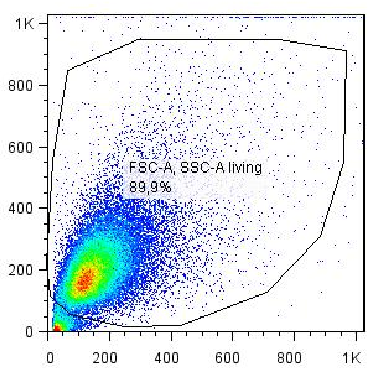

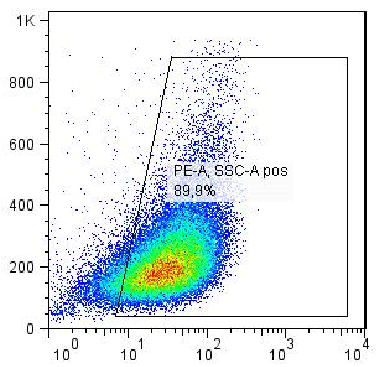

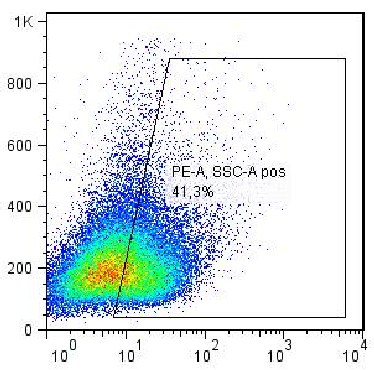


α-SMA

SSC

FSC

SSC

CD54

SSC

CD90

SSC


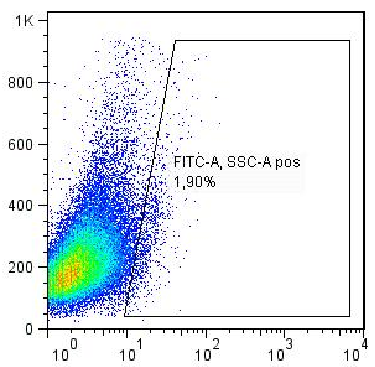

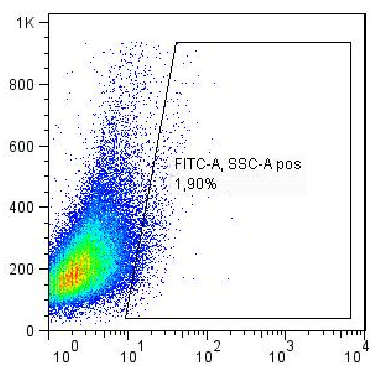

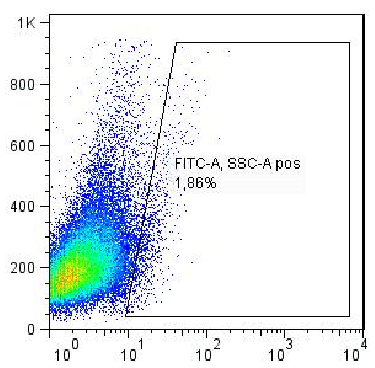


CD34

SSC

CD45

SSC

CD31

SSC


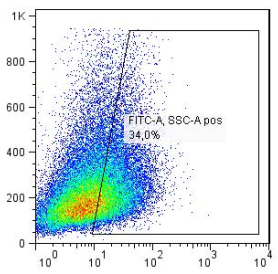

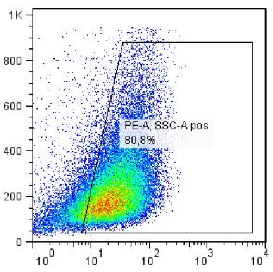

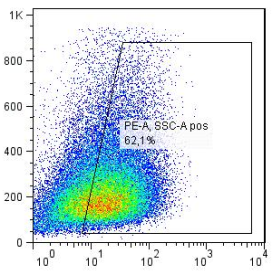

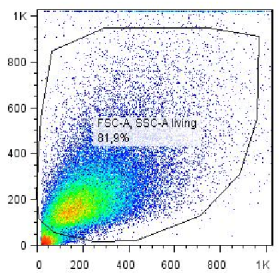
**Cancer-associated lung fibroblasts:**

α-SMA

SSC

CD90

CD54

SSC

SSC

FSC

SSC


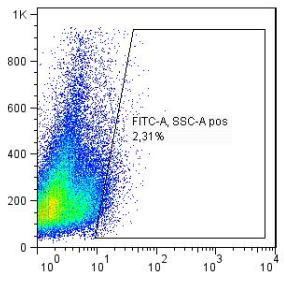

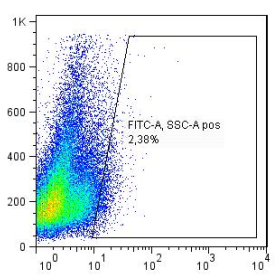

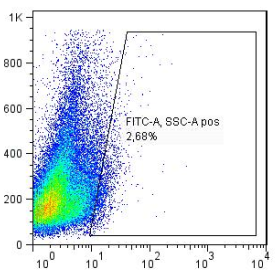


CD34

SSC

CD31

SSC

CD45

SSC

**Figure S1**
