# Supplementary material for: Plasticity of fibroblasts demonstrated by tissue-specific and function-related proteome profiling
Source: Clin Proteomics. 2014 Nov 21;11(1):41. doi: 10.1186/1559-0275-11-41 (PMC4448269; doi:10.1186/1559-0275-11-41)
Supplement: Supplementary file 13 — Additional file 13: Table S12: emPAI values for all proteins listed in Figure 2. For each protein the emPAI values determined by us in the different sub-cellular fractions (sn, cell supernatant; cyt, cytoplasmic fraction; nuc, nuclear fraction) of the respective cell type and cell state are indicated. AccNr, Swiss-Prot accession number. (DOCX 18 KB) [file 12014_2014_89_MOESM13_ESM.docx]

**Table S 12**

| **AccNr** | **Protein name** | **Skin fibs** | **Skin fibs + IL1b** | **Melanoma-assoc. fibs** | **NHLF** | **NHLF + IL1b** | **Lung Cancer-assoc. fibs** | **BM fibs** | **BM fibs + IL1b** | **MM-assoc. fibs** |
| --- | --- | --- | --- | --- | --- | --- | --- | --- | --- | --- |
| Q07507 | Dermatopontin | 1.130sn  0.52cyt | 0.780sn | 0.233sn | - | - | - | - | - | - |
| O75094 | Slit homolog 3 protein | 0.063nuc | 0.050nuc | - | - | - | - | - | - | - |
| P09619 | PDGFR-beta | 0.056cyt | 0.041cyt | 0.041sn | - | - | - | - | - | 0.105cyt  0.041nuc |
| Q8WWI1 | LIM domain only protein 7 | 0.016cyt  0.076nuc | 0.084nuc | - | - | - | - | 0.056nuc | 0.068nuc | 0.228nuc |
| Q12884 | Seprase/ FAP | 0.065cyt | - | - | - | - | - | 0.065cyt | 0.182cyt | 0.087cyt |
| P19320 | VCAM-1/ CD106 | - | - | - | - | - | - | 0.151cyt | 0.099cyt | 0.182cyt |
| Q92954 | Proteoglycan 4 | - | - | - | - | - | - | 0.026sn | 0.017sn | 0.017sn |
| Q12929 | EGF receptor kinase substrate 8 | - | - | - | - | - | - | 0.063nuc | - | 0.070nuc |
| P36222 | Chitinase 3-like protein 1 | - | - | - | - | - | 0.166sn | 0.946sn | 0.825sn  0.080nuc | - |
| Q8WWM9 | Cytoglobin | - | - | - | 0.533cyt | 0.370cyt | 0.166cyt | 0.166cyt | 0.585cyt | 0.421cyt  0.166nuc |
| P17900 | Ganglioside GM2 activator | - | - | - | 0.194cyt  0.425sn | 0.425cyt | - | 0.348cyt | - | 0.309cyt |
| P29279 | CTGF | - | - | - | 0.148sn | 0.086sn | 0.086sn | 0.086sn | 0.086sn | 0.179sn  0.086cyt  0.086nuc |
| O75787 | Renin receptor | - | - | - | 0.424sn | 0.682sn | 0.668sn | - | 0.214sn | - |
| Q9BUD6 | Spondin-2 | - | - | - | 0.763sn | 0.244sn | 0.797sn | - | - | - |
| O00468 | Agrin | - | - | - | 0.048sn | 0. 048sn | - | - | - | - |
| Q8N474 | Secreted frizzled-related protein 1 | - | - | 0.129sn | 0.202sn  0.129nuc | - | 0.202sn | - | - | - |
| P03956 | MMP-1/ fibroblast collagenase | 0.315sn | 0.315sn | 0.136sn | 0.468sn | 1.443sn  0.136nuc | - | - | - | - |
