# Supplementary material for: Plasticity of fibroblasts demonstrated by tissue-specific and function-related proteome profiling
Source: Clin Proteomics. 2014 Nov 21;11(1):41. doi: 10.1186/1559-0275-11-41 (PMC4448269; doi:10.1186/1559-0275-11-41)
Supplement: Supplementary file 14 — Additional file 14: Table S13: emPAI values for all proteins listed in Table 1. For each protein the emPAI values determined by us in the different sub-cellular fractions (sn, cell supernatant; cyt, cytoplasmic fraction; nuc, nuclear fraction) of the respective cell type and cell state are indicated. AccNr, Swiss-Prot accession number. (DOCX 22 KB) [file 12014_2014_89_MOESM14_ESM.docx]

**Table S13**

| **AccNr** | **Protein name** | **Skin fibroblasts** | | **NHLF** |  | **BM fibroblasts** |  |
| --- | --- | --- | --- | --- | --- | --- | --- |
| **Supernatant:** |  | Control | IL-1β-treated | Control | IL-1β-treated | Control | IL-1β-treated |
| P05231 | IL-6 | _ | 0.965sn | _ | 0.359sn | _ | 0.818sn |
| [P10145](http://www.uniprot.org/uniprot/P10145) | Il-8 | _ | 2.749sn  0.389cyt  0.389nuc | _ | 1.036sn  0.931cyt | _ | 0.570sn  0.389cyt |
| P09341 | GRO-alpha/CXCl1 | _ | 1.826sn | _ | 1.967sn  1.783cyt  0.292nuc | _ | 1.783sn |
| P80162 | CXCL6 | _ | 0.931sn | _ | 0.660sn  1.036cyt  1.307nuc | _ | 0.660sn |
| P42830 | CXCL5 | _ | 0.660sn | _ | 0.660sn  0.931cyt  0.389nuc | _ | 0.660sn |
| P08254 | MMP-3/Stromelysin-1 | _ | 0.816sn  0.150cyt | _ | 0.520sn | _ | _ |
| P07585 | Decorin | 0.086sn  0.086cyt | 1.304sn  0.179cyt  0.258nuc | 0.699sn  0.086cyt  0.086nuc | 1.031sn  0.086cyt  0.086nuc | 0.179sn | 0.389sn |
| P08253 | MMP-2 | 0.137sn | 0.560sn | 0.317sn  0.105cyt | 0.286sn  0.051cyt  0.051nuc | 0.137sn | 0.366sn |
| P09603 | CSF-1 | _ | 0.389sn | 0.179sn | 0.280sn | _ | 0.086sn |
| P98066 | TSG-6 | _ | 0.315sn | _ | _ | _ | 0.444sn  0.233nuc |
| P26022 | PTX3/ TSG-14 | 0.632sn | 0.939sn  0.413nuc | _ | 0.499sn | 1.148sn  0.585cyt | 1.225sn  0.830cyt  0.264nuc |
| P19875 | GRO-beta /CXCL2 | _ | 1.075sn | _ | _ | _ | _ |
| P05121 | PAI-1 | 0.526sn | 0.363sn  0.093cyt  0.093nuc | 0.194sn | 0.759sn  0.093cyt  0.093nuc | 0.361sn  0.093cyt | 0.781sn  0.093cyt |
| O00391 | Sulfhydryl oxidase 1 | (0.044sn) | 0.066sn | 0.619sn  0.186cyt | 0.348sn  0.105cyt  0.089nuc | _ | 0.186sn |
| Q15063 | Periostin | (0.040sn) | _ | 0.130sn | 0.173sn  0.062cyt | (0.040sn) | 0.126sn  0.040cyt  0.083nuc |
| Q9Y240 | SCGF | 0.274sn | _ | (0.129sn) | 0.624sn | _ | _ |
| **Cytoplasm:** |  |  |  |  |  |  |  |
| O00469 | PLOD-2 | (0.044cyt) | 0.066cyt | _ | 0.154cyt | (0.044sn) | 0.299sn |
| P05362 | ICAM-1 (CD54) | _ | _ | (0.072cyt  0.072nuc) | 0.072cyt  0.072nuc | _ | 0.150cyt |
| [P05120](http://www.uniprot.org/uniprot/P05120) | PAI-2 | 0.229cyt | 0.931cyt  0.179sn | _ | 0.381cyt  0.179sn | 0.179cyt | _ |
| **Nuclear Extract:** |  |  |  |  |  |  |  |
| Q8WX93 | Palladin | 0.055nuc | 0.239nuc | 0. 027cyt  0. 093nuc | 0. 041cyt  0. 155nuc | 0.027cyt  0.070nuc | 0.200nuc |
| P51911 | Calponin-1 | 0.658nuc | 0.650nuc | 0. 163nuc | 0.864nuc | 0.222nuc | 0. 421nuc |
| Q16666 | γ-IFN-inducible protein 16 | 0.062nuc | 0.055nuc  0.040cyt | 0. 055nuc | 0. 062nuc | 0. 055nuc | 0. 177nuc |
